# Assessment of self-medication practices with antibiotics among undergraduate university students in Rwanda

**DOI:** 10.11604/pamj.2019.33.307.18139

**Published:** 2019-08-19

**Authors:** Jacques Tuyishimire, Funmbi Okoya, Adebisi Yusuff Adebayo, Fabrice Humura, Don Eliseo Lucero-Prisno III

**Affiliations:** 1 Faculty of Pharmacy, University of Rwanda, Huye, Rwanda; 2 Department of Pharmaceutical Services, Lagos State Ministry of Health, Nigeria; 3 Department of Pharmacy, University of Ibadan, Nigeria; 4 International Pharmaceutical Federation, the Hague, Netherlands; 5 Department of Health and Environmental Sciences, Xi’an Jiaotong-Liverpool University, Suzhou,China; 6 Department of Global Health and Development, London School of Hygiene and Tropical Medicine, United Kingdom

**Keywords:** Antibiotics, self-medication, education, university students

## Abstract

**Introduction:**

Antimicrobial resistance (AR) is on a rise as one of the major global public health threats. It is therefore important to assess contributory factors to the rise in the cases of resistance reported. The main objective of this study was to assess the self-medication practices with antibiotics among the University of Rwanda students in Huye Campus.

**Methods:**

A sample of 570 students from all levels and colleges of the University of Rwanda in Huye Campus were selected using a simple random sampling to participate in this study. A questionnaire was administered to be answered individually by the consented respondents where the self-medication practices with antibiotics in the past 6 months were assessed. The results were statistically analyzed using SPSS v.16.

**Results:**

The study showed that 12.1% (n=69) practiced self-medication with antibiotics. The major reason for self-medication with antibiotics was illness not serious to have a consultation (50.72%). The main diseases being treated were common cold/fever/cough (47.83%). The most used antibiotic for self-medication was Amoxicillin capsules (59.42%), while the main source of antibiotics was the community pharmacy (72.42%).

**Conclusion:**

Self-medication with antibiotics is not uncommon among the university students. Regarding the main reasons of self-medication with antibiotics, diseases being treated, and the antibiotics used, it was found that all these may be related to the students' lack of knowledge about the need for rational use of antibiotics and a study was needed to confirm it.

## Introduction

Globally, self-medication was found to be on the rise [1]. Self-medication has been defined as "the taking of drugs, herbs or home remedies on one's own initiative, or on the advice of another person, without consulting a doctor" [2]. Major problems related to self-medication include wastage of resources, increased resistance of pathogens (which generally cause serious health hazards such as adverse reactions), prolonged suffering and drug dependence [3]. Among other considered medications being used without a prescription, in developing countries, are antibiotics [4]. Self-medication, particularly with antibiotics, has been widely encouraged by unregulated supply chains. This may increase the risk of inappropriate use and the selection of resistant bacterial strains [5, 6]. Self-medication may be helpful in facilitating the recovery from some minor illnesses. However, risks and benefits should be weighed reasonably as some serious problems may be due to the self-use of medications [7]. Medicines allowed for self-medication are those called Over-the-counter (OTC) drugs, and they can be dispensed without a doctor's prescription through pharmacies, mostly in the less developed countries [8]. Over the counter (OTC) drugs are a form of self-medication as the buyer diagnoses their own illness and buys a specific drug to treat it [4]. OTC drugs provide symptom relief for conditions that do not always require medical intervention. Self-medication has been used widely to combat behavioral and psychological problems such as smoking. Pharmacists can help patients to choose the right OTC smoking cessation products to help them successfully quit smoking. Up to 300,000 people each year are able to reduce their risk of lung cancer, emphysema, stroke, heart attack, and complications in pregnancy because of self-care products, which are readily available. For example, non-prescription nicotine helps people to quit smoking [9]. Self medication has certain advantages as it is convenient, economical, and reduces wastage of medical resources on minor illnesses. However, as the disease recognized may not be correct, there is a delay in meeting a health care worker; the side effects of the medication are not known; inappropriate usage of antibiotics can lead to drug resistance; and taking the same drug with other drugs can lead to drug interactions and sometimes, drug addiction [10, 11]. Among the people who practice self-medication, particularly with antibiotics, some researchers have shown that university students are included [12]. This study aimed to assess the self-medication practices with antibiotics among, the University of Rwanda students in Huye Campus.

## Methods

The study was conducted among undergraduate students of the University of Rwanda (UR), HUYE Campus. This Campus is located in the Southern Province of Rwanda, HUYE District. The campus was selected because it has all colleges of UR except College of Education. Those colleges are: College of Medicine and Health Sciences (CMHS), College of Science and Technology (CST), College of Agriculture and Veterinary Medicine (CAVM), College of Business and Economics (CBE) and College of Arts and Social Sciences (CASS). The study was a descriptive study, and it consisted of a structured survey done among the university students, where a structured paper questionnaire consisting of close-ended and open-ended questions was shared to be completed by all the respondents. Answers from the questionnaires were exclusively used as the data source. Sample size was calculated using Yamane formula, where sample size should be 384 students. However, 570 students were used in this study as respondents, which is 186 students above the calculated sample size. A sample of 570 students from all colleges of the UR in Huye Campus was selected to participate in this study using simple random sampling. A questionnaire was administered to be answered individually by the consented respondents, where self-medication with antibiotics in the past 6 months was assessed. The results were statistically analyzed using SPSS v.16, and the results were shown in frequency and percentages.

**Participants' inclusion criteria:** this study included people who fulfill the following criteria: must be a registered undergraduate student of UR; must have been physically located in the UR for at least 6 months; must consent to participate in this research project.

**Participants' exclusion criteria:** this study didn't take into consideration: people who were not UR/Huye campus undergraduate students; people who haven't been in the university for up to 6 months; and people who didn't consent to participate in the study. Regarding the permission to conduct this research, a research proposal was presented to the Department of Pharmacy in UR, HUYE Campus requesting a recommendation addressed to the dean of students in the campus to collect data from the university students. The participants' informed consent to the study was also required and was signed by the respondents as a proof of their will to participate. The participants answered the questionnaires individually and could withdraw at anytime or choose not to complete the questionnaire.

## Results

A total of 570 students were enrolled in the study, which included 45.09% male and 54.91% female students. Colleges represented were CBE (39.30%), CST (26.14%), CMHS (17.37%), CASS (11.05%) and CAVM (5.26%). The predominant age of the participants was between 21 and 25 years, ranging from 18 to above 30 years. The results highlighted that 12.1% (n=69) of the responding students had self-medicated with antibiotics in the last 6 months (mid-October 2015 to mid-March 2016). Self-medication was more frequent among the female respondents compared to the male respondents (14.70% vs. 8.95%, female and male respondents respectively; Table 1). The findings highlight that the greatest proportion of students practiced self-medication with antibiotics (SMA) because they thought the illness was not serious at 50.72% (n=35), followed by assumed medical knowledge at 15.94% (n=11), lack of time for consultation due to class lectures at 14.49% (n=10), following doctor's advice on previous encounter at 10.14% (n=7), and discomforting attitude of the health care providers at 2.90% (n=2). (The reasons for self-medication are highlighted in Table 2) The main diseases treated with SMA are common cold/flu/cough at 47.83% (n=33), sore throat at 14.49% (n=10), diarrhea at 13.04% (n=9), skin infections at 13.04%(n=9), and eye infections at 1.45% (n=1),headache 2.90% ( n=2), and wound 1.45% (n=1). (Table 3 highlights diseases being treated with SMA) The common antibiotics used in SMA are amoxicillin capsules (see Table 4) at 59.42% (n=41) , tetracyclines at 2.90% (n=3) and others like chloramphenicol tablets, ciprofloxacin tablets, neo-medrol cream, nitrofurantoin tablets, oracefal tablets, penicillin capsules, streptomycin capsules and bactrim tablets were hardly used. The main source of antibiotics (Table 5) used in self-medication is community pharmacy with 72.46% (n=50), friends with 13.04% (n=9), and leftover medicines at 7.25% (n=5).

**Table 1:** Distribution of self-medication with antibiotics by gender

Self-medication by gender	Self-medication percentages (frequency)	
	Not self-medicated	Self-medicated
Female (n=313)	85.30% (267)	14.70% (46)
Male (n=257)	91.05% (234)	8.95% (23)

**Table 2:** Reasons for self-medication with antibiotics

Reasons for Self Medications with Antibiotic	Percentage (Frequency)
Illness not serious to have consultation	50.72% (35)
Assumed medical knowledge	15.94% (11)
Lack of time for consultation due to class lectures	14.49% (10)
Following doctor's advice on previous encounter	10.14% (7)
Discomforting attitude of the health care providers	2.90% (2)
Not mentioned	5.80% (4)

**Table 3:** Conditions where students self medicate with Antibiotics

Diseases being treated	Percentage (Frequency)
Common cold/fever/cough	47.83% (33)
Sore throat	14.49% (10)
Diarrhea	13.04% (9)
Skin infections	13.04% (9)
Eyes infections	1.45% (1)
Headache	2.90% (2)
Wound	1.45% (1)
Not mentioned	5.80% (4)

**Table 4:** Antibiotics used in self medications

Name of the antibiotics	Dosage form	Percentage (Frequency)
Amoxicillin	Capsules	59.42% (41)
Tetracyclines	Tablets	2.90% (2)
Chloramphenicol	Tablets	1.45% (1)
Ciprofloxacin	Tablets	1.45% (1)
Neo-Medrol	Cream	1.45% (1)
Nitrofurantoin	Tablets	1.45% (1)
Oracefal	Tablets	1.45% (1)
Penicillin	Capsules	1.45% (1)
Streptomycin	Tablets	1.45% (1)
Bactrim	Tablets	1.45% (1)
Not mentioned	(-)	26.09% (18)

**Table 5:** Sources of antibiotics used in self-medication

Sources of antibiotics	Percentage (Frequency)
Community Pharmacy	72.46% (50)
Friends	13.04% (9)
Leftover Medicines	7.25% (5)
Not mentioned	7.25% (5)

## Discussion

With prevalence of 12.1% (n=69), data from this study indicates that irrational use of antibiotics is practiced among university students. This proportion is less to that reported among tertiary level students in Accra [13], where the prevalence of SMA stood at 70%. By comparison, the prevalence of SMA among tertiary level students reported 53.5% in Nigeria [14], 76% in Pakistan [15], and 45% in Iran [16]. Studies have also found that educated people have a greater tendency to practice self-medication than illiterates. Higher levels of education and professional status have been associated with and considered as a predictive factor for self-medication [17, 18]. The study shows that female students engaged in self-medication with antibiotics more than male students. The differences in frequencies of self medication between gender agrees with a study done among tertiary level students in Accra, Ghana, in which the prevalence of self medication with antibiotics was 67% and 73%, Male and female respectively. It is suggested that the difference may be partly due to the female monthly menstrual cycle during which there are hormonal changes and relatively low immunity. At such times in their cycle, females may feel reluctant to visit the hospital and hence their higher frequency of self medication. This is partly confirmed by a study in Nigeria which showed that 25% of women employed antibiotics to treat menstrual symptom [13, 14]. However, the relationship between gender and self-medication was not assessed by this study. Nevertheless, other studies showed that self-medication patterns are influenced by age, gender, education level, family, society, medical knowledge, perception of illness, self-care orientation, and drug advertisements [19]. Regarding the reasons of self-medication with antibiotics, majority (50.72%) practiced SMA because they thought the illness was not serious and results are in line with another research conducted among the university students of Karachi [20], where it is said that the main reason for SMA was "illness not serious" and other shown reasons in this study. This main reason of SMA showed that students might think an illness is not serious while it is. This may be related to the lack of knowledge about health concerns, particularly the rational use of antibiotics. Findings are comparable with a study done among Chinese university students where some likely reasons for high SMA rates in China were misconceptions about antibiotic use for flu-like symptoms, false confidence in antibiotic use, high medical costs at health care centers, long waiting hours for outpatient services in hospitals and clinics, lack of trust in prescribing physicians, easy access to antibiotics, and profit-driven persuasion by community pharmacists [21].

The high extent of self-medication by students could be due to their belief that it would provide quick relief from illness. That was also seen in a study done by Angamo and Wabe in 2012 [22]. The use of antibiotics was high in all illness categories including conditions for which antibiotics were not recommended by WHO [23], such as common cold and diarrhea. This finding was similar to studies from Southwestern Nigeria. The study done on self-medication with antibiotics, among the university students revealed that antibiotics were used for respiratory diseases [12]. However, these diseases might have not been well treated through SMA because the students didn't follow the health care delivery process as a whole. This might result to the exacerbation of the disease or other health complications. Misuse of antibiotics is due to diagnostic uncertainty, lack of prescriber knowledge, lack of opportunity for follow-up, easy availability of antibiotics, and lack of treatment guidelines [24]. Majority of the respondents (59.42%) used Amoxicillin capsules while doing self-medication. The reasons for choosing Amoxicillin as an antibiotic in SMA were not identified by this study. However, many reasons can be attributed to choosing antibiotics. Thus, further studies should be carried out to confirm the choice criteria. The main source of antibiotics used in self-medication was community pharmacy (72.46%). These results confirm the study done in different places, but particularly among the university students in Karachi [20], where antibiotics used in self-medication was 41.4% and the main source was community pharmacies [25, 26]. The community pharmacies contributed a lot to SMA by dispensing the non-prescribed antibiotics. This concerns the pharmacy profession regulation enforcements, as students would not have gotten antibiotics if the community pharmacies refused to dispense them without a prescription [13]. It would be indispensable to embrace strict antibiotic guidelines which have shown affirmative outcome. A good example is the Chilean Ministry of Health, which has strictly restricted the purchase of antibiotics without medical prescription since 1999. This action resulted in a 43% decrease in antibiotic use in the outpatient setting [27], which represents an impressive result. Our study had some limitations as it exclusively considered students that live within the UR/HUYE Campus, without considering the students who live outside the campus or in nearby regions. Also, the UR Huye Campus includes all colleges except CE, which is located in Kigali.

## Conclusion

Our study observed self-medication with antibiotics among students in University of Rwanda/ Huye Campus. Educating the university students more about the dangers of self-medication with antibiotics and rational use of antibiotics may be adequate to stop self-medication with antibiotics. In addition to strengthening the regulation on dispensing antibiotics without prescriptions is essential, as the community pharmacies are the major source of antibiotics used in self-medication with antibiotics.

### What is known about this topic

- Prevalence of self-medication is likely to be influenced by the knowledge of medications;
- Self-medication is a common practice in university students.

### What this study adds

- Our study suggests that female students practice SMA more than males and another study proved this relationship;
- The community pharmacies were found to be the main source of antibiotics used in self-medication. Had they refused to dispense the antibiotics without a prescription, SMA would have been considerably reduced.

## Competing interests

The authors declare no competing interests.

## References

[1] Rohit K Verma, Lalit Mohan, Manisha Pandey (2010). Evaluation of self-medication among professional students in North India: Proper statutory drug control must be implemented. Asian J Pharm Clin Res.

[2] Hernandez-Juyol M, Job-Quesada JR (2002). Dentistry and self-medication: a current challenge. Med Oral.

[3] James H, Handu SS, Khalid AJ, Khaja AI, Otoom S, Sequeira RP (2006). Evaluation of the knowledge, attitude and practice of self-medication among first-year medical students. Med Princ Pract.

[4] World Health Organization (2000). Guidelines for the regulatory assessment of medicinal products for use in self-medication. WHODEDM/QSM/001.

[5] Kunin CM (1993). Resistance to antimicrobial drugs--a worldwide calamity. Ann Intern Med.

[6] Grigoryan L (2007). Is self-medication with antibiotics in Europe driven by prescribed use. Journal of Antimicrobial Chemotherapy.

[7] Lawan UM, Abubakar IS, Jibo AM, Rufai A (2013). Pattern, awareness and perceptions of health hazards associated with self-medication among adult residents of kanometropolis, northwestern Nigeria. Indian J Community Med.

[8] Pwar NV, Jain SK, Sahi SR (2009). Self-medication: how safe: Ask to your pharmacist. The pharma review.

[9] You JH, Wong FY, Chan FW, Wong EL, Yeoh EK (2011). Public perception on the role of community pharmacists in self-medication and self-care in Hong Kong. BMC Clin Pharmacol.

[10] Calabresi P, Cupini LM (2005). Medication-overuse headache: similarities with drug addiction. Trends Pharmacol Sci.

[11] Greenhalgh T (1987). Drug prescription and self-medication in India: an exploratory survey. Soc Sci Med.

[12] Osemene KP, Lamikanra A (2012). A Study of the Prevalence of Self-Medication Practice among University Students, Southwestern Nigeria. Tropical Journal of Pharmaceutical Research.

[13] Eric S, Donkor, Patience B, Tetteh-Quarcoo, Patrick Nartey, Isaac O, Agyeman (2012). Self-Medication Practices with Antibiotics among Tertiary Level Students in Accra, Ghana: a cross-sectional Study. Int J Environ Res Public Health.

[14] Sapkota AR, Coker ME, Goldstein RER, Atkinson NL, Sweet SJ, Sopeju PO, Ojo MT, Otivhia E, Ayepola OO, Olajuyigbe OO, Shireman L, Pottinger PS, Ojo KK (2010). Self-medication with antibiotics for the treatment of menstrual symptoms in southwest Nigeria: a cross sectional study. BMC Public Health.

[15] Syed NZ, Reema S, Sana W, Akbar Z, Talha V, Mehrine S (2008). Self-medication amongst university students of Karachi: prevalence, knowledge and attitudes. J Pak. Med Assoc.

[16] Sarahroodi S, Arzi A, Sawalha AF, Ashtarinezhad A (2010). Antibiotics self-medication among southern Iranian university students. Int J Pharmacol.

[17] Klemenc-Ketis Z, Hladnik Z, Kersnik J (2010). Self-medication among healthcare and non-healthcare students at University of Ljubljana, Slovenia. Med Princ Pract.

[18] Martins AP, Miranda Ada C, Mendes Z, Soares MA, Ferreira P, Nogueira A (2002). Self-medication in a Portuguese urban population: a prevalence study. Pharmacoepidemiology Drug Saf.

[19] Afolabi AO (2008). Factors influencing the pattern of self medication in an adult Nigerian population. Ann Afr Med.

[20] Yasmin Mumtaz, Ashraf Jahangeer, Tahira Mujtaba, Shahla Zafar, Sara Adnan (2011). Self Medication among University Students of Karachi. Journal of Liaquat University of Medical and Health Sciences.

[21] Zhu X, Pann H, Yang Z, Cui B, Zhang D, Ba-Thein W (2016). Self-medication practices with antibiotics among Chinese university students. Public Health.

[22] Angamo MT, Wabe NT (2012). Knowledge, attitude and practice of self medication in Southwest Ethiopia. International Journal of Pharmaceutical Sciences and Research.

[23] World Health Organization (2007). Pocket book of hospital care for children guidelines for the management of common illnesses with limited resources.

[24] World Health Organization (2010). Medicines: rational use of medicines.

[25] Togoobaatar G, Ikeda N, Ali M, Sonomjamts M, Dashdemberel S, Mori R, Shibuya K (2010). Survey of non-prescribed use of antibiotics for children in an urban community in Mongolia. Bull World Health Organ.

[26] Syed Jawad Shah (2014). Self-medication with antibiotics among non-medical university students of Karachi: a cross-sectional study. BMC Pharmacology and Toxicology.

[27] Bavestrello L, Cabello A, Casanova D (2002). Impact of regulatory measures in the trends of community consumption of antibiotics in Chile. Rev Med Chil.

